# Using Discounting Biases, Risk Characteristics, and Perceived Control Improves Preventive Programs

**Published:** 2007-06

**Authors:** Monica Ortendahl

**Affiliations:** *Royal Institute of Technology, Stockholm, Sweden*

**Keywords:** prevention, framing, messages, time, risk, control

## Abstract

Health promotion often works toward remote goals with a trade-off between costs today and benefits in the future. However, for individuals using a positive discount rate for health outcomes a healthy state many years ahead has such a small value that it is difficult to motivate them to engage in preventive behaviors. The framework of time and risk for analysis can perform a useful role in health education and information where the framing of different features of risk might diminish discounting and increase motivation to change behavior. Personal versus general risk and perceived control related to preventive programs are discussed. A summary of valuation factors in preventive programs based on literature review is presented: (a) long-term decisions are sensitive to discount rates; (b) discount rates vary by level of uncertainty, individuals, and contexts; (c) personal risks from adverse health behaviors are judged as smaller than the same risks for people in general; (d) probability discounting is used, if the risk is perceived as controllable; (e) people’s tendency to discount future consequences might be suppressed by lowering the amount of perceived control.

## INTRODUCTION

A 30-year-old female cigarette smoker has been a smoker for ten years. She is now pregnant and believes she has control and could quit whenever she wants. She recognizes that quitting smoking yields several benefits for mother and child alike, and that smoking will likely lead to serious health problems like emphysema and lung cancer in 30 or 40 years. Despite this prognosis, she decides that she does not want to quit now, as she believes that these health problems will occur to others but not to her.

This common scenario is usually interpreted as a problem of addiction. Another perspective is that she has a high discount rate for future health, and thus events decades in the future do not influence current decisions. From this perspective, a central health education goal would be to decrease her discount rate and to redefine her health goal as limiting lifetime disability. Other examples where the time dimension is important are normalized blood glucose to prevent late vessel complications (1), investment in calcium intake or vitamin D to prevent fractures because of osteoporosis (2), and cholesterol lowering to prevent coronary heart disease (3).

Broad-based health and preventive programs have been initiated (4-6) with the anticipation that these educational and information projects would produce positive results in terms of individual behavior change. However, subsequent evaluations of the programs indicate that overall program effects are modest in size and often temporary (7, 8).

This well-established problem has tentatively been explained by a discrepancy in time perspectives for health promotion goals and individual behavior (9, 10). Health promotion often works toward remote goals, and many preventive health decisions include a trade-off between costs today and benefits in the future. However, the benefits often occur so far in the future that they may seem of little value to the individual relative to the immediate costs.

In our era of chronic illness, with antecedents often distant in time health behavior is frequently influenced in a significant manner by the principles and practice of discounting (11). For individuals using a positive discount rate for health outcomes a healthy state many years ahead has such a small value that it is difficult to engage them in preventive behaviors (12, 13). As discounting is related to risk it requires that uncertainty is analyzed being an unavoidable condition in preventive efforts (14). The impact of time and risk on many judgments and decisions has increasingly been recognized as an important factor in the health field, as it has become evident that many situations concern the exchange of present-day costs for future benefits (15).

The framework of time and risk for analysis can perform a useful role in health education and information, where the framing of different features of risk might diminish discounting. A method based on framing effects could be used in all countries, especially those with middle and lower economies, as it does not lead to any increased costs. On the contrary, it is cost-effective, as it could reduce the incidence and prevalence of many diseases with consequent costs for individual and society. In the present article time and risk related to preventive programs are discussed with a special focus on how discounting biases for a particular risk should be taken into consideration when framing health messages. The impact of personal versus general risk and perceived control on framing of health messages is also discussed. The criteria for selection of articles were potential practical application when formulating health messages.

### Time and Risk

Time discounting processes vary with individuals and contexts. Therefore, no single model is expected to describe discounting processes completely. In societal health policy and education future costs and health benefits are devalued at a rate of approximately 3-5 percent annually using the same rate for both money and health (16). Individual decision makers, in contrast, frequently use much higher annual discount rates in the range of 50 percent to 100 percent, where discounting biases appear more prevalent in health than in economic decisions (17). This discrepancy between discount rates for health policy and individuals could lead individuals to reject a health message recommended by health planners and educators.

Most existing work on inter-temporal choice has largely been restricted to a trade-off between two outcomes of varying values and over different periods. The role of risk does not seem to have been studied to the same extent, although it is known that risk generally has a large impact on judgments and decisions being made, either by the individual or by health educators (18). A decision will often have to be made when to undertake a risk-reducing behavior, rather than whether to undertake this behavior. A similar judgment is also required when a preventive procedure should be adopted. Introducing features of risk and time in health messages gives a more complete, if more complex picture of variables that should influence health education and policy.

However, risk is not an easy concept and, therefore, it is of importance that risk information is presented to be used as a sound basis for decisions implying preventive procedures (19). This should include information about absolute as well as relative risk, as they are perceived differently (20, 21). Absolute and relative risks, however, are statistical concepts, and further problems arise when psychological estimates of risk constitute the basis for behavior change.

Discount rates have been found to differ for different types of risk, and it has been suggested that the varying results might partly be explained by a difference in the value of risk reduction (22). Empirical work on risk reduction has earlier mostly focused on risk in the present time or it has used a-temporal models. However, when it comes to risks, proposed time discounting utility-based theories of the value of life have increasingly included both a-temporal and inter-temporal life-cycle models, in which the timing of risks is relevant (10).

### Personal Versus General Risk

A need in health information is to understand the impact of psychological factors like personal versus general risk, where personal risk relates to oneself and general risk to others. Personal risks from adverse health behaviors have been found to be judged as smaller than the same risks for people in general (23). If allowed to indulge in speculation, the time variable should have a larger impact on personal risks compared with public risks, as it to a larger extent might be related to emotive components. However, this area has not been thoroughly and systematically investigated.

The difference found between personal and general risk has been related to perceived control (24), implying that when people believe they have control over a risk-filled situation, they judge their personal risk to be smaller than general risks. Presumably, this is because people do not consider others as equally capable or willing to protect themselves from risk. It could be contended that with a perceived low personal risk the engagement and motivation to change behavior is smaller.

A speculative conclusion suggested here is that primary prevention and secondary prevention are perceived differently, depending on perceived controllability. In primary prevention no health problems have still been obtained, and the control is perceived as high, whereas in secondary prevention the individual suffers from for example coronary heart disease, and as a consequence perceives a lower control. A lower perceived control may give a lower discount rate with a higher compliance to health programs. However, it is of importance to prevent the occurrence of cardiovascular disease in the first place, and to inform about risk and loss of control due to smoking, over-eating and not exercising to decrease discount rate and to change behavior to a more healthy life. Future studies on health promotion need to take this into account.

Time discounting can be in value, in probability, or in both (12). Regardless of whether expected value is discounted or not, probability discounting is used if the risk is perceived as controllable (25). Therefore, for perceived controllable risks probability seems to represent a more general mechanism than value discounting. People’s tendency to discount future consequences might be suppressed by lowering the amount of perceived control, a circumstance to be considered when framing health messages.

It could be speculated that perception of decreased control, paradoxically, may lead to greater exercise of control. If factors are emphasized that oppose the idea that temporal delay will result in increased control, the discount rate might be lowered. For example, with smoking-related behaviors, the possibilities for future corrective actions are limited because of the detrimental effects from smoking; some adverse effects of smoking, such as emphysema, are irreversible.

## DISCUSSION

It could be summarized that different features of risk and time constitute a basis in framing health messages. Discounting for future events could be based on an event in the future having less importance than one in the present, where the adverse outcome may have different weights.

Overall, likelihood of a specific adverse outcome is a parameter affecting the estimate of future risk and its consequences. However, severity may not be the same for everyone who experiences the event. One example is occupational back pain being relatively mild in some persons but disabling in others (26). Therefore, it seems that specific individual factors modify risk, and person-specific modifiers are likely distributed differently in time.

Another parameter is temporal distribution of risk not being homogenous throughout the work life-span of the individual. When risks with different time profiles compete a trade-off in timing of risk has been found (27), which also has to be taken into account when framing health messages. A trade-off in timing of risk will then occur rather than a trade-off of risk for money, related to the present value of risk reduction that takes place in the future. Risk has been studied in many settings, and evidence suggests that risk-risk trade-offs are more stable than responses to risk-money choices (28).

For health education programs that reduce long-term health risks, the market view implies that less emphasis is placed on reducing long-term health risks in favor of a greater emphasis on programs that achieve reductions that are more rapid in health risks. This conclusion has been found valid (29) even if those programs may save fewer statistical lives in the long run.

For some risks, the median discount rate may be close to the real market discount rate, whereas for other risks it may not. Consequently, we can reject the hypothesis that the discount rates for all risks are equal. This means that health messages should be framed in different ways for different risks to obtain a low a discount rate, as a low devaluation of future outcomes increases the possibility for a change in behavior.

Differences in valuation between money and health have been documented (30) with higher discount rate for health than for money. As time discounting effects are discrepant, and cannot easily be generalized between the two fields of money and health, problems arise if treating death and other health effects as monetary equivalents. Therefore, the necessity of deferred gratification in health decisions is a central issue to be included in health messages, where the expected benefits are reductions in the probability of morbidity and mortality from diseases in the future.

Another issue in framing messages is how resources are allocated over time, as it has an impact on mortality and morbidity, where morbidity and mortality have different costs (31). If your risk of death declines now, you gain some lifetime (32). Yet, when does the gain occur? Does it occur at the end of your expected life-span or before risk reduction? Alternatively, does it even occur now at the very moment of risk reduction?

Fries has proposed the Compression of Morbidity paradigm emphasizing delaying the onset of morbidity, and thereby shortening its duration, with the intent to reduce lifetime illness and morbidity (33). The paradigm envisions reduction of lifetime infirmity and of medical care costs by compression of the period of morbidity between an increasing average age at onset of disability and the average age of death. In senior populations fractures associated with osteoporosis are a major cause of morbidity. Preventive interventions aimed at reducing the age-specific incidence of fracture are crucial in reducing the morbidity resulting from these fractures. The effect of preventive interventions is likely to postpone the age of onset of morbidity.

Another time-risk interaction in this context is the optimum timing of different preventive actions. Therefore, by introducing risk for certain outcomes, the relationship between morbidity and mortality might be conceptually extended. The years added by preventive measures may not be purely healthy ones, suggesting that the quantitative aspects of these years are of importance. The relationship between risk for certain outcomes and morbidity and mortality is important as the majority of deaths under age 65 are preventable, including premature diseases, injuries and other types of morbidity.

As is evident from the discussion above many choices in health both at an individual and societal level involve decisions with a trade-off between something now and something later (34). The evaluation of health risk reduction, routine preventive care, and population screening programs include health measures that improve future health (35). Inter-temporal choices, which imply many medical situations on a societal level like preventive programs on smoking depend, in part, on the exchange of present-day costs for future benefits (36, 37). However, for the individual the value of not smoking vs. smoking is small because of the acquisition of the benefits (or the disadvantages of the former) takes place in the remote future.

Moreover, time profiles might be supposed to vary with reference to people’s age, gender, race, creed, color, and ethnic group. Another issue is whether the considerations apply to different countries, for example those under unfavorable economic conditions. These issues seem to have focused upon to a lesser extent and need further attention.

## CONCLUSIONS

The intriguing relationship between the time dimension and the risk dimension has been discussed, and it is suggested that this relationship has applications to preventive medicine. Introducing features of risk related to time gives a more complex description of variables that influence the framing of health messages (see Table 1).

**Table 1:** Valuation factors in preventive programs

Long-term decisions are sensitive to discount rates.
Discount rates vary by level of uncertainty, individuals, and contexts.
Personal risks from adverse health behaviors are judged as smaller than the same risks for people in general.
Probability discounting is used, if the risk is perceived as controllable.
People’s tendency to discount future consequences might be suppressed by lowering the amount of perceived control.

Time and risk considerations are of salience for individual choices, as well as health education choices that have an impact on long-term health outcomes. The effect of time and risk has not extensively been taken into account in attempts to explain health proceedings, and when allocating resources these factors must be included to perform a complete analysis of decisions. A decision analysis model based on time of outcome and chance of outcome is a theoretical and practical approach that has an intuitive appeal when it comes to health education.

To be concluded, problems related to maintenance of behavior change are an important challenge to disciplines dealing with health-related behaviors. Perhaps one reason for the lack of conclusive data in this field of research is that models used in explaining individual behavior assume that people behave rationally, once they have obtained reliable, credible and factual information. Health education programs in the future may benefit from considering the importance of discounting biases and risk characteristics. In these efforts we need to integrate research from medicine, economics, psychology and education. Further work on the validity of this approach should be an important priority.
